# Supplementary material for: Influence factors on the critical micelle concentration determination using pyrene as a probe and a simple method of preparing samples
Source: R Soc Open Sci. 2020 Mar 4;7(3):192092. doi: 10.1098/rsos.192092 (PMC7137975; doi:10.1098/rsos.192092)
Supplement: The molecular structures of surfactants, experimental detailed procedures and Figures S1-14 have been uploaded as electronic supplementary material. [file rsos192092supp1.pdf]

Electronic Supplementary Material (ESM)

Influent factors on pyrene-based CMC determination and a simple method of preparing samples

Hao Li,[†] Danna Hu,[†] Feiqing Liang, Xiaowei Huang and Qiuhua Zhu[*]

Content

Molecular structures of surfactants SDS/CTAB/CHAPS/Triton X-100/ BS-12 2

Factors influencing the CMC determination of SDS using pyrene as probe (Figures S1–6) 2

Procedures of preparing samples by method I 2

Procedures of preparing samples by method II 2

Procedures of preparing samples by method III 3

Determination of the CMC values of CTAB/CHAPS/Triton X-100/ BS-12 from samples prepared by Method I (Figures S7 and S8)...7

Determination of the CMC values of CTAB/CHAPS/Triton X-100/ BS-12 from samples prepared by Method III (Figures S9 and S10) 8

CMC determination of SDS'obtained from different supplier(Figures S11–14) 9

CMC determination of SDS' from samples prepared by method I.............................9

CMC determination of SDS' from samples prepared by method II 9

CMC determination of SDS′ from samples prepared by method III 10

CMC determination of SDS′ by conductive method... 10

Molecular structures of surfactants SDS/CTAB/CHAPS/Triton X-100/ BS-12

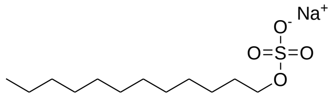

$C_{12}H_{25}NaO_4S$
Mol. Wt.: 288.38
Sodium dodecyl sulfate (SDS)

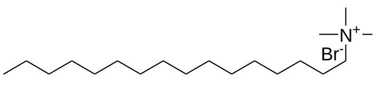

$C_{19}H_{42}BrN$
Mol. Wt.: 364.45
Cetyltrimethylammonium bromide (CTAB)

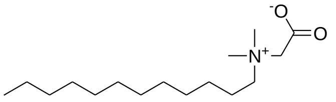

$C_{16}H_{33}NO_2$
Mol. Wt.: 271.44
Dodecyl dimethyl betaine (BS-12)

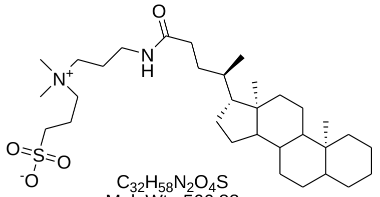

$C_{32}H_{58}N_2O_4S$
Mol. Wt.: 566.88
3-[(3-cholamidopropyl)dimethylammonio]-1-propanesulfonate (CHAPS)

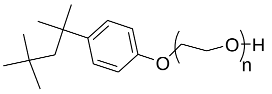

$C_{34}H_{62}O_{11}$ n≈10
Mol. Wt.:646.85
Polyethylene glycol tert-octylphenyl ether (Triton X-100)

Factors influencing the CMC determination of SDS using pyrene as probe (Figures S1–6)

Procedures of preparing samples by method I

a) Preparation of concentrated SDS solution (about 2 CMC) containing a certain amount of pyrene

A certain amount of surfactant stock solution and pyrene stock solution were added into a 100 mL volumetric flask, shaking well and keeping at least for 30 min before filling these flasks to the mark with water to prepare a concentrated surfactant solution with a certain concentration of pyrene(0.2–1 μM).

b) Preparation of a series of SDS solutions with different concentrations containing a certain amount of pyrene

As shown in the table below, different volumes of the concentrated SDS solution (10 mM) prepared in procedure a were added into different 5 mL volumetric flasks, filling these flasks to the mark with water containing the same concentration of pyrene as that in the concentrated SDS solution (10 mM).

entry	1	2	3	4	5	6	7	8	9	10	11	12	13	14
c_{SDS}/ mM	4	5	5.6	6	6.6	7	7.4	7.6	8	8.4	8.8	9.2	9.6	10
V_{SDS}/ mL	2	2.5	2.8	3	3.3	3.5	3.7	3.8	4	4.2	4.4	4.6	4.8	5

Procedures of preparing samples by method II

a) Preparation of pyrene saturated aqueous solution

Excessive amount of pyrene and double distilled water were added into a 1000 mL reagent bottle, ultrasounded for about 20 minutes, and stored at room temperature at least 24 hours.

b) Preparation of concentrated SDS solution (20 mM)

20.00 mL of SDS stock solution (100 mM) was added into a 100 mL volumetric flask, filling the flask to the mark with water and shaking well.

c) Preparation of a series of SDS solutions with different concentrations containing a certain amount of pyrene.

Procedures of preparing samples by method III

The procedures of method III are the same as those of method I except diluting the concentrated SDS solution containing a certain of pyrene into different concentrations of SDS solutions with pure water rather than water containing the same concentration of pyrene as that in the concentrated SDS solution.

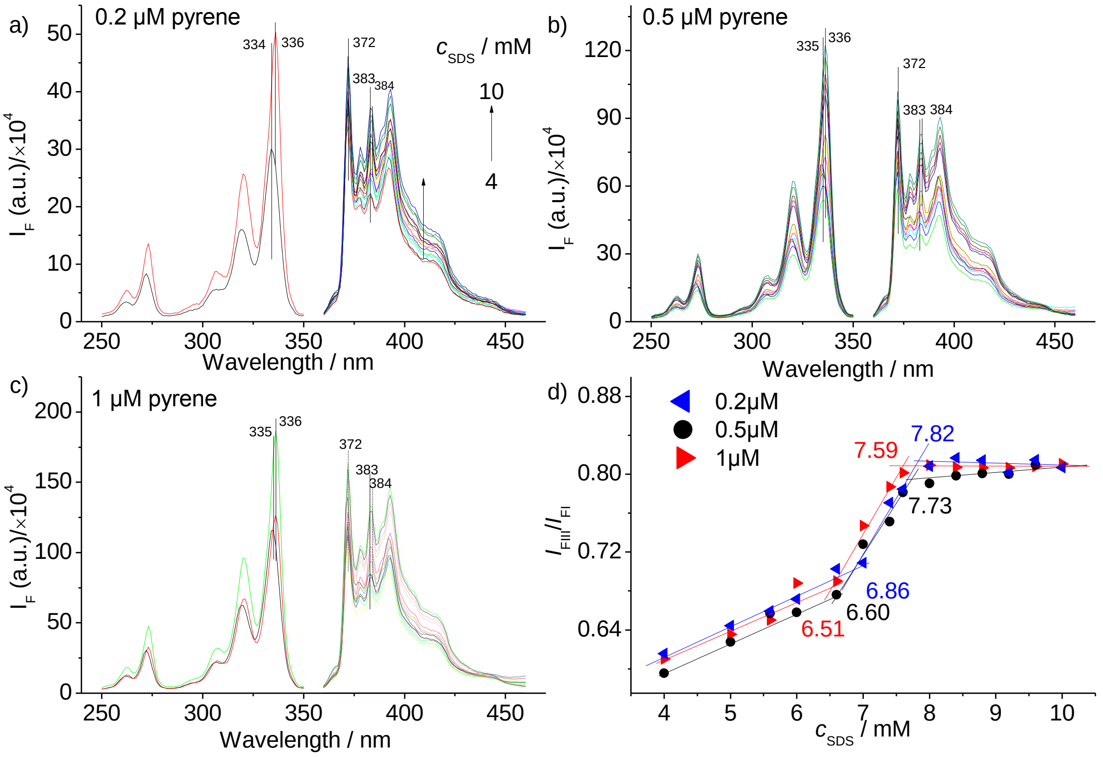

Figure S1. Influence of the concentration of pyrene on the CMC value of SDS. a) to c) Excitation (left) and emission (right) spectra of SDS solutions containing 0.2, 0.5 and 1 μM of pyrene, respectively. d) Relationship between the I_{FIII}/I_{FI} value of pyrene and the concentration of SDS. Slit widths: 2 and 2 nm. Samples were prepared by method I and determined immediately.

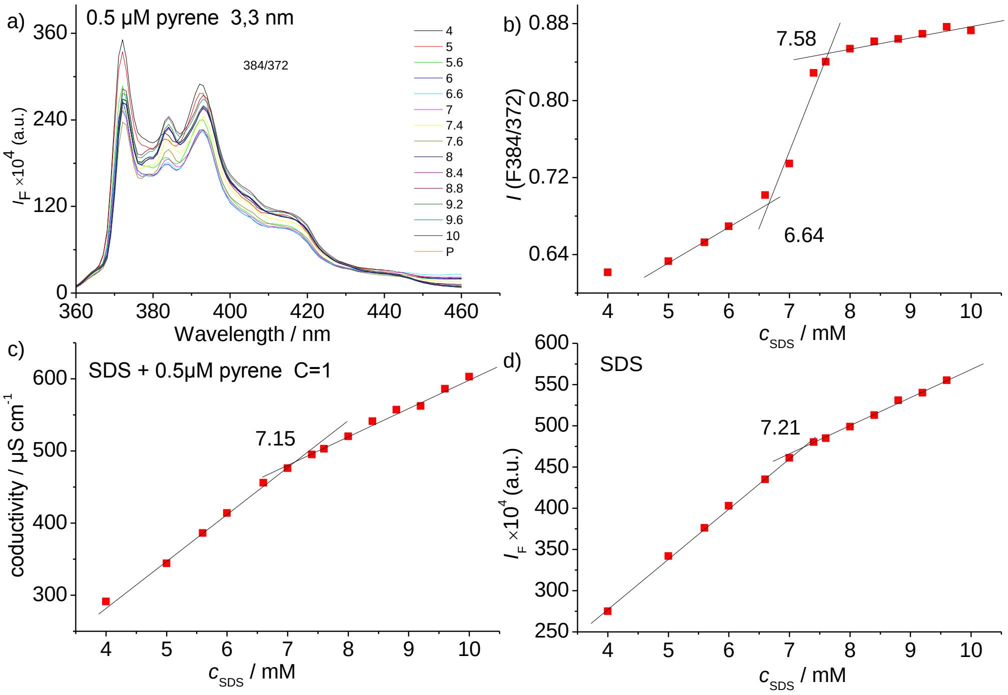

Figure S2. SDS solutions with different concentrations (4–10 mM) in the presence (a–c) and absence (d) of pyrene (0.5 μM).a) Emission spectra of pyrene; b) Relationship between SDS concentration and the I_{FIII}/I_{FI} of pyrene in a); c) and d) Relationship between SDS concentration and the conductivity of SDS solutions with and without pyrene, respectively. Samples were prepared by method I.

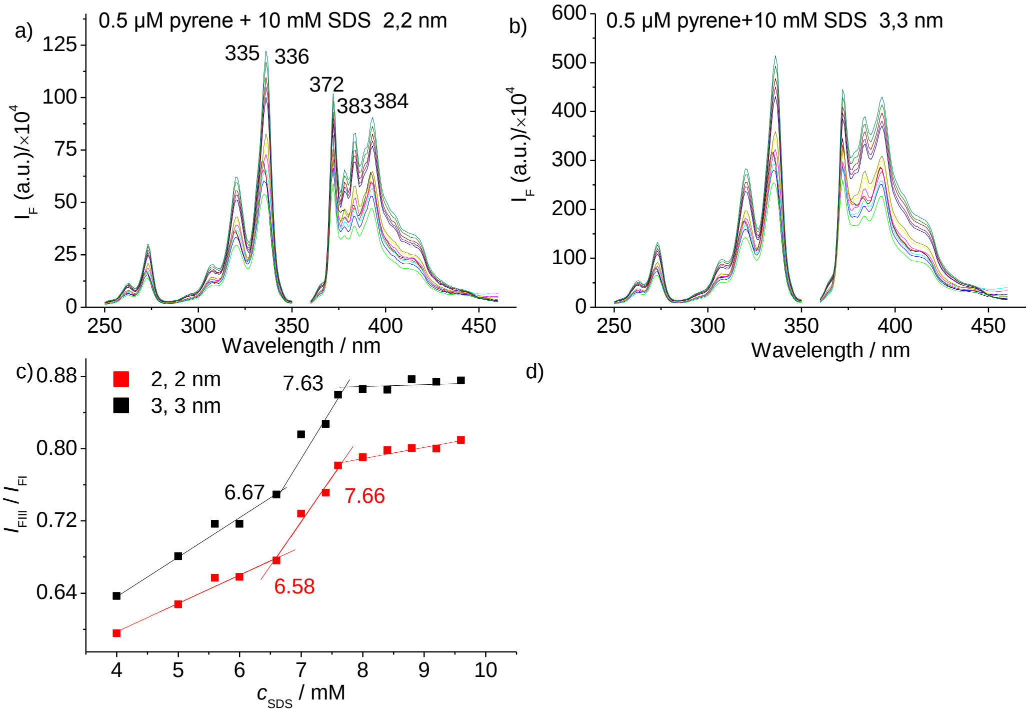

Figure S3. Influence of instrument slit widths on the I_{FIII}/I_{FI} values of pyrene. a) and b) Excitation (left) and emission (right) spectra of SDS solutions with 0.5 μM of pyrene measured at 2, 2 nm and 3, 3 nm slit widths, respectively. d) Relationship between the I_{FIII}/I_{FI} value and SDS concentration. Samples were prepared by method I.

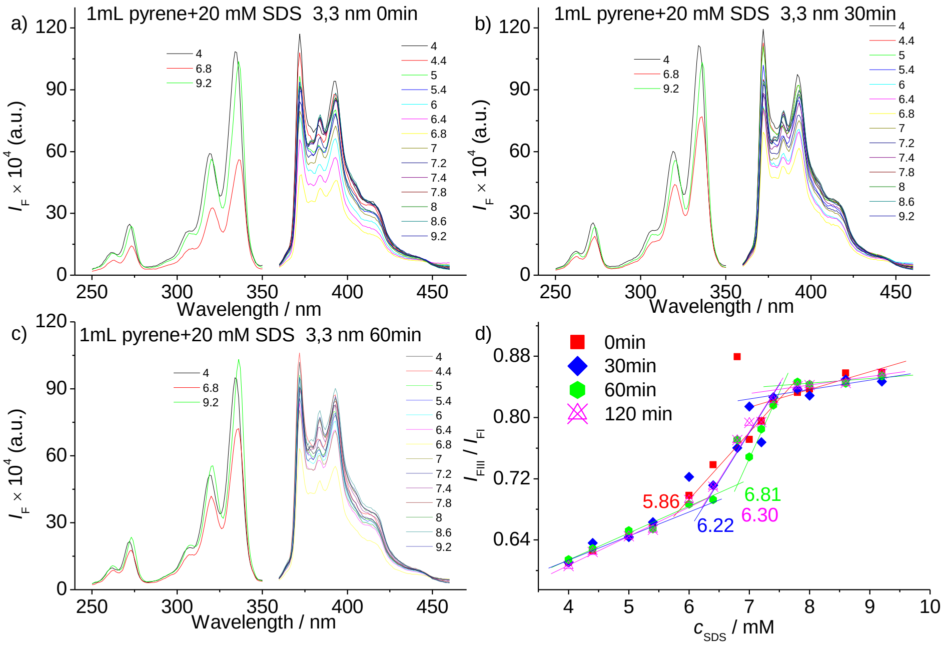

Figure S4. Influence of time before diluting samples on CMC determination of SDS (Samples were prepared by method II:adding 1 mL of pyrene saturated water solution into different 5 mL volumetric flasks containing different volumes of 20 mM SDS solution, shaking well, keeping 0, 30, 60 and 120 min, then filling the flasks to the mark with water and determining immediately.). a) to c) Excitation and emission spectra of pyrene in samples kept 0, 30 and 60 min, respectively, before diluting samples. d) Relationship between SDS concentration and the I_{FIII}/I_{FI} value of pyrene in samples kept 0, 30, 60 and 120 min before diluting, respectively.

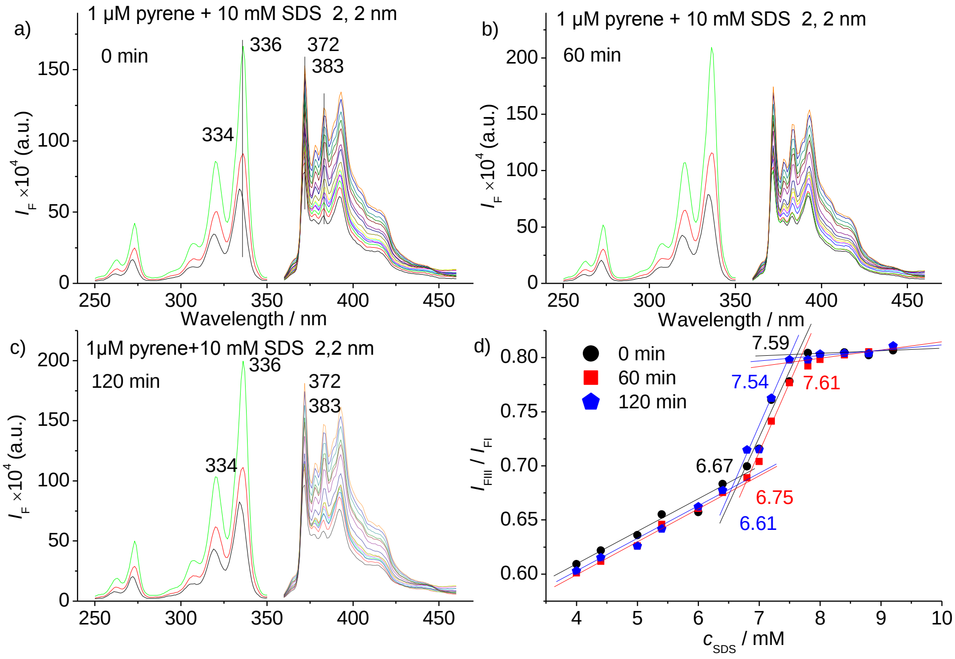

Figure S5. Influence of time before diluting samples on CMC determination of SDS (Samples were prepared by method III: 10 mM SDS concentrated solutions with 1 μM pyrene

were prepared by adding 200 μL of 0.5 mM pyrene ethanol stock solution and 10 mL of 100 mM SDS stock solution into three 100 mL volumetric flasks, shaking well, kept 0, 60 and 120 min, respectively, before filling the flasks to the mark with water. Then different volumes of the SDS concentrated solutions ware added into different 5 mL flasks, filling the flasks to the mark with water and determining immediately.).a) – c) Excitation (left) and emission (right) spectra in samples kept for 0, 60 and 120 min, respectively. d）Relationship between SDS concentration and the I_{FIII}/I_{FI} value of pyrene in samples kept 0, 60 and 120 min before diluting, respectively.

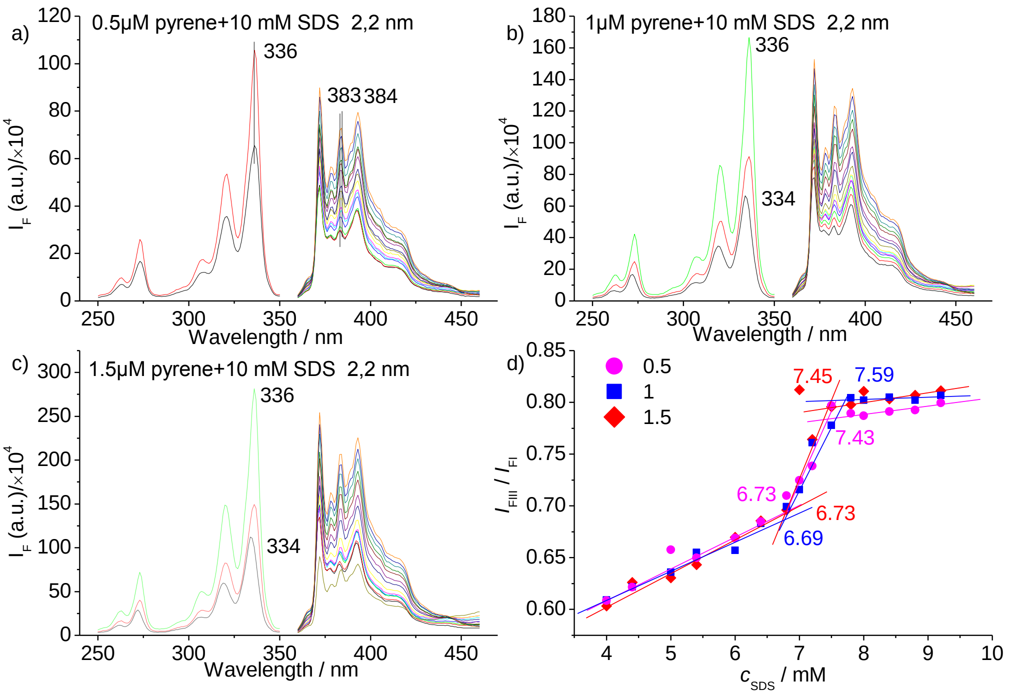

Figure S6. Influence of the concentration of pyrene on CMC determination. a) to c) Excitation (left) and emission (right) spectra of SDS solutions containing 0.5, 1 and 1.5 μM of pyrene, respectively. d) Relationship between I_{FIII}/I_{FI} value and SDS concentration. Samples were prepared by method III.

Determination of the CMC values of CTAB/CHAPS/Triton X-100/ BS-12 from samples prepared by Method I (Figures S7 and S8)

Samples were prepared by diluting concentrated CTAB (2 mM), CHAPS (12.5 mM), Triton X-100 (0.4 mM) and BS-12 (5 mM) containing 0.5 μM pyrene into a series of samples with different concentrations of surfactant and 0.5 μM pyrene using water containing 0.5 μM pyrene.

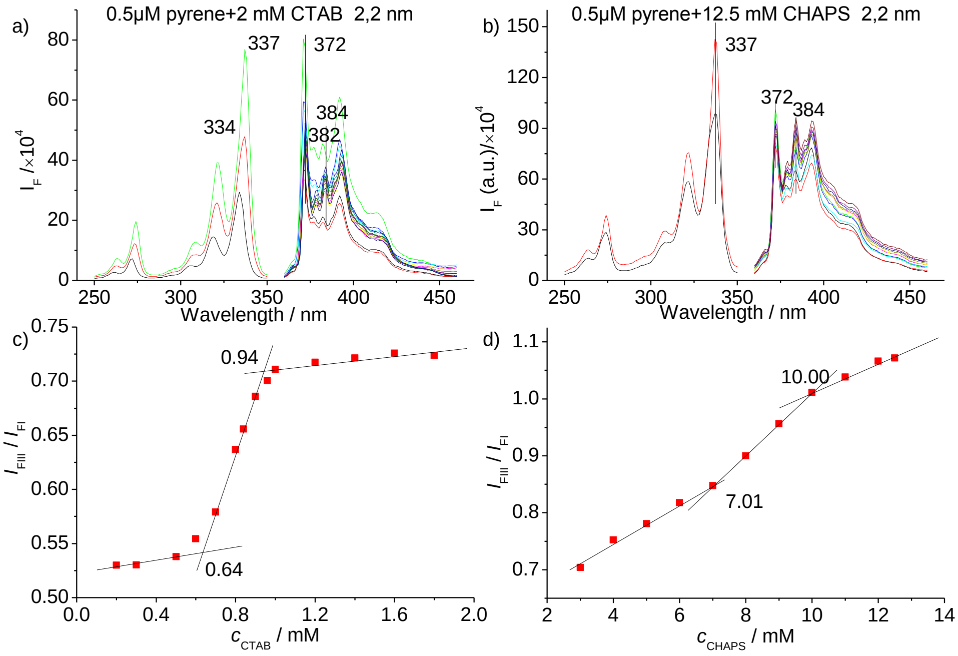

Figure S7. CMC determination of cationic CTAB and zwitterionic CHAPS from samples prepared by method I. a)/b) Excitation (left) and emission (right) spectra of the samples prepared by diluting 2/12.5 mM CTAB/CHAPS containing 0.5 μMpyrene, respectively. c)/d) Relationship between I_{FIII}/I_{FI} value and the concentration of CTAB/CHAPS.

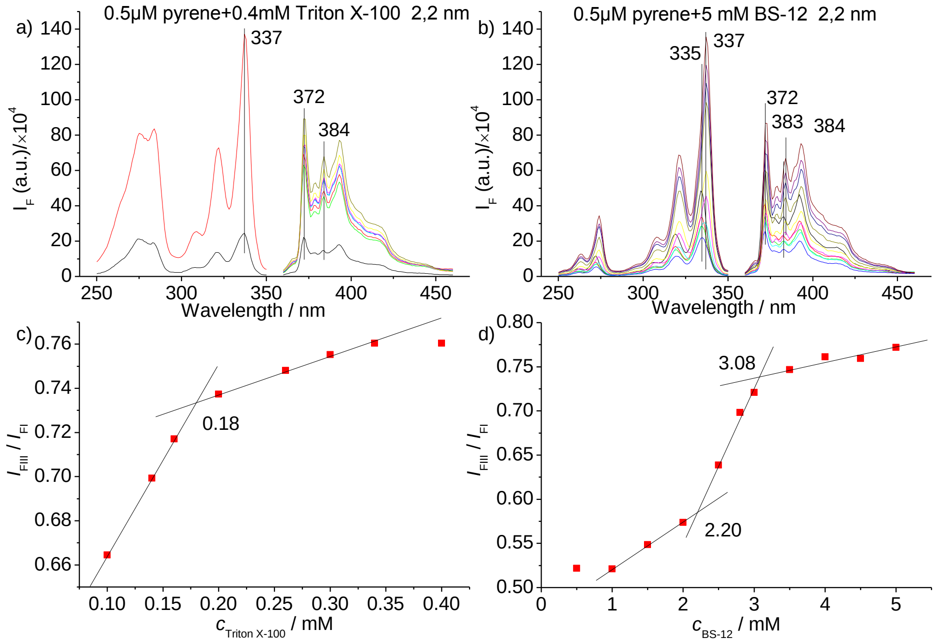

Figure S8. Determination of the CMC values of Triton X-100/ BS-12 from samples prepared by method I. a)/b) Excitation (left) and emission (right) spectra of the samples prepared by

diluting 0.4/5 mM Triton X-100/ BS-12 containing 0.5 μMpyrene, respectively. c)/d) Relationship between I_{FIII}/I_{FI} value and the concentration of Triton X-100/ BS-12.

Determination of the CMC values of CTAB/CHAPS/Triton X-100/ BS-12 from samples prepared by Method III (Figures S9 and S10)

Samples were prepared by diluting concentrated CTAB (2 mM), CHAPS (12.5 mM), Triton X-100 (0.4 mM) and BS-12 (5 mM) containing 1μM pyrene into a series of samples with different concentrations of surfactant and pyrene using water.

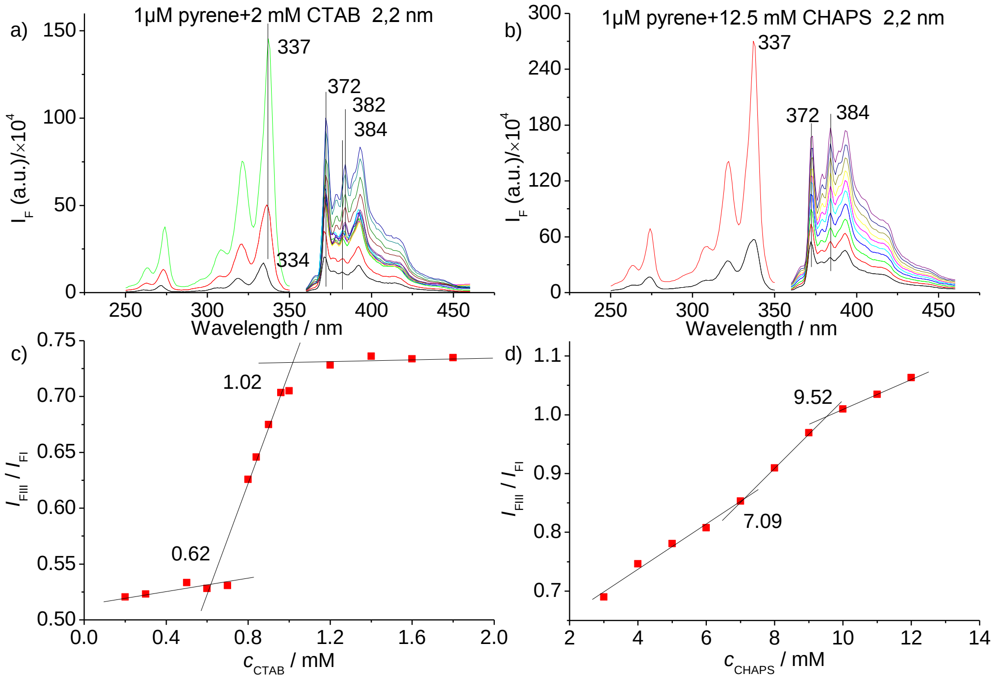

Figure S9. Determination of the CMC values of CTAB/CHAPS via method III. a)/b) Excitation (left) and emission (right) spectra of the samples prepared by diluting 2/12.5 mM CTAB/CHAPS containing 1 μM pyrene, respectively. c)/d) Relationship between I_{FIII}/I_{FI} value and the concentration of CTAB/CHAPS.

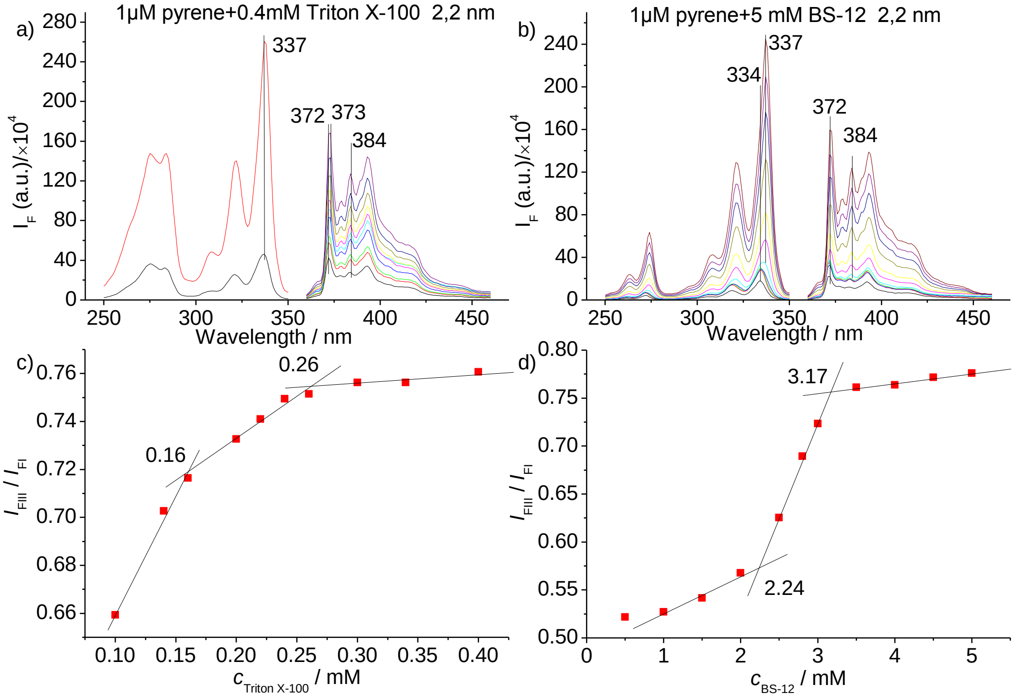

Figure S10. Determination of the CMC values of Triton X-100/ BS-12 via method III. a)/b) Excitation (left) and emission (right) spectra of the samples prepared by diluting 0.4/5 mM Triton X-100/ BS-12 containing 1 µM pyrene, respectively. c)/d) Relationship between I_{FIII}/I_{FI} value and the concentration of Triton X-100/ BS-12.

CMC determination of SDS'obtained from different supplier(Figures S11–14)

CMC determination of SDS' from samples prepared by method I

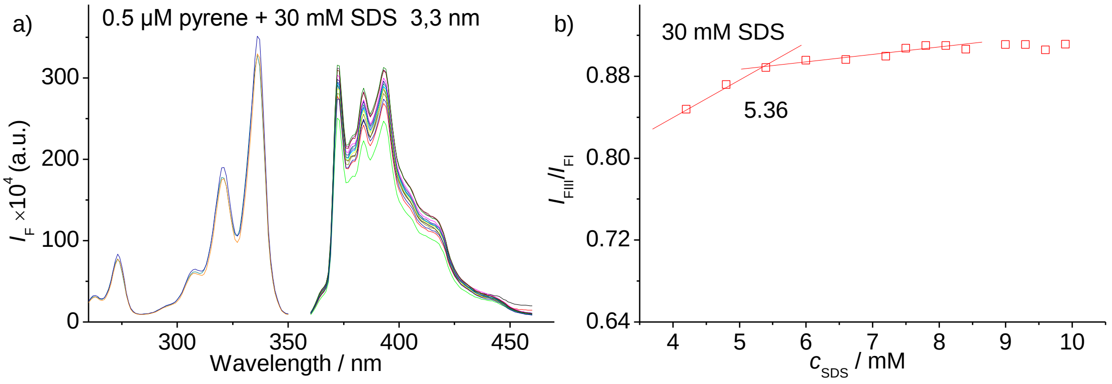

Figure S11. CMC determination of SDS′ using pyrene as probe and method I to prepare samples. (a) Excitation (left) and emission (right) spectra of pyrene (0.5 µM) in different concentrations of SDS′ (4 – 10 mM). (b) Relationship between I_{FIII}/I_{FI} value and the concentration of SDS′.

CMC determination of SDS' from samples prepared by method II

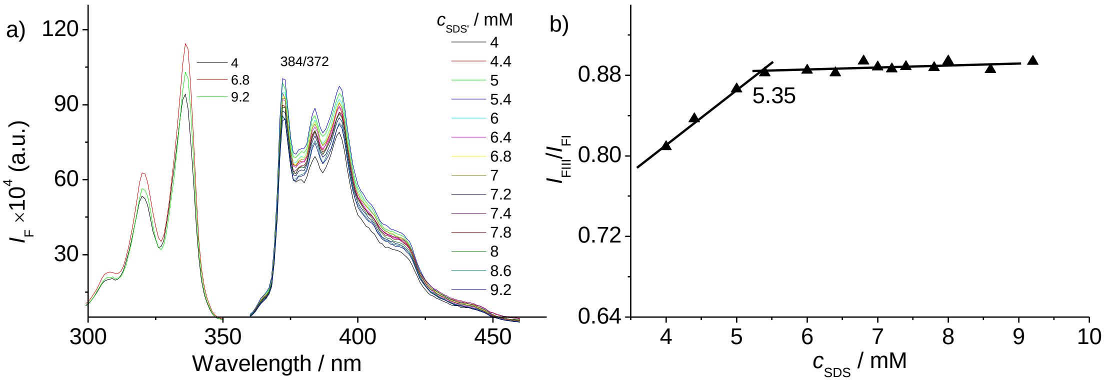

Figure S12. CMC determination of SDS′ using pyrene as probe and method II to prepare samples. (a) Excitation (left) and emission (right) spectra of pyrene (1 mL pyrene saturated water solution) in different concentrations of SDS′ (4 – 10 mM). (b) Relationship between I_{FIII}/I_{FI} value and the concentration of SDS′.

CMC determination of SDS′ from samples prepared by method III

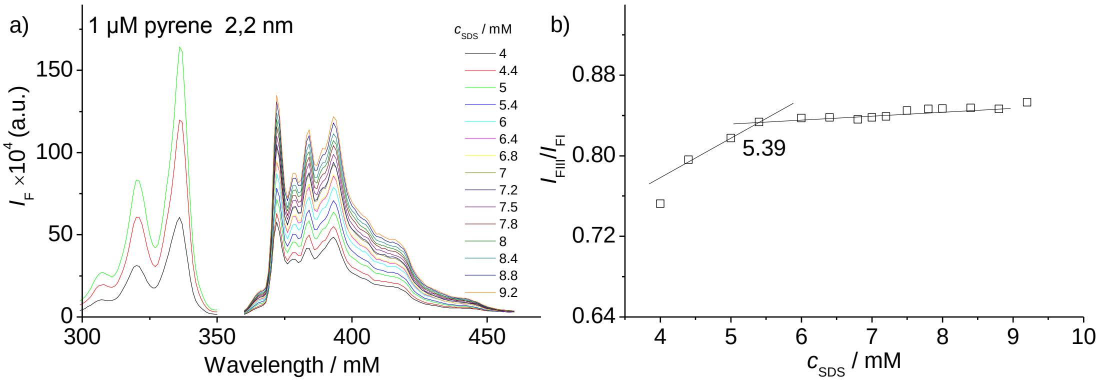

Figure S13. CMC determination of SDS′ using pyrene as probe and method III to prepare samples. (a) Excitation (left) and emission (right) spectra of pyrene(0.4–1 μM) in different concentrations of SDS′ (4 – 10 mM). (b) Relationship between I_{FIII}/I_{FI} and the concentration of SDS′.

CMC determination of SDS′ by conductive method

Preparation of samples:

a) Preparation of concentrated SDS′ solution (10 mM)

Adding 0.1442g SDS′（Meryer）to a 50mL volumetric flask, then filling the flask to the mark using water.

b) Preparation of different concentrations of SDS′ solution

As shown in the table below, different volumes of the concentrated SDS′ solution (10 mM) prepared in procedure a were added into different 5 mL volumetric flasks using 5 mL graduated pipette, then filling the flask to the mark using water.

entry	1	2	3	4	5	6	7	8	9	10	11	12	13
c_{SDS}/ mM	4	5	5.6	6	6.6	7	7.4	7.6	8	8.4	8.8	9.2	9.6
V/ mL	2	2.5	2.8	3	3.3	3.5	3.7	3.8	4	4.2	4.4	4.6	4.8
μS cm^{-1}	329	392	426	450	478	499	520	531	548	562	587	599	621

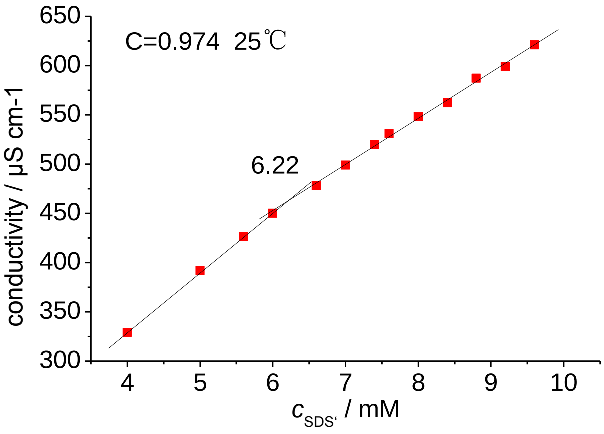

Figure S14. Relationship between the concentration and conductivity of SDS′ solutions.
